# Clinical application value of metagenomic next-generation sequencing in the diagnosis of central nervous system infections

**DOI:** 10.3389/fbioe.2023.885877

**Published:** 2023-04-11

**Authors:** Ying Liu, Weiwei Zhu, Mengfan Jiao, Wenhu Guo, Yonggang Luo

**Affiliations:** ^1^ Gene Hospital of Henan Province, The First Affiliated Hospital of Zhengzhou University, Zhengzhou, China; ^2^ Department of Infectious Diseases, The First Affiliated Hospital of Zhengzhou University, Zhengzhou, China; ^3^ Agene Medical Laboratory, Fuzhou, China; ^4^ School of Medical Technology and Engineering, Fujian Medical University, Zhengzhou, China; ^5^ Department of Intensive Care Unit, The First Affiliated Hospital of Zhengzhou University, Zhengzhou, China

**Keywords:** metagenomic next-generation, cerebrospinal fluid, central nervous system infections, diagnosis, conventional detection

## Abstract

**Objectives:** The purpose of this study is to evaluate the clinical application value of metagenomic next-generation sequencing (mNGS) in central nervous system (CNS) infections.

**Methods:** Both mNGS and routine examination of cerebrospinal fluid (CSF) samples from patients with CNS infections retrospectively analyzed the efficacy of mNGS in this cohort and were ultimately compared with a clinical diagnosis.

**Results:** A total of 94 cases consistent with CNS infections were included in the analysis. The positive rate for mNGS is 60.6% (57/94), which is significantly higher than 20.2% (19/94; *p* < 0.01) detected using conventional methods. mNGS detected 21 pathogenic strains that could not be detected by routine testing. Routine tests were positive for two pathogens but negative for mNGS. The sensitivity and specificity of mNGS in the diagnosis of central nervous system infections were 89.5% and 44%, respectively, when compared with traditional tests. At discharge, 20 (21.3%) patients were cured, 55 (58.5%) patients showed improvements, five (5.3%) patients did not recover, and two (2.1%) patients died.

**Conclusion:** mNGS has unique advantages in the diagnosis of central nervous system infections. mNGS tests can be performed when patients are clinically suspected of having a central nervous system infection but no pathogenic evidence.

## Introduction

CNS infections in the brain and spinal cord, including encephalitis, meningitis, myelitis, and brain abscesses, are mainly caused by not only bacteria and viruses but also a few pathogens such as fungi and parasites. Diagnosis of bacterial meningitis is difficult, and it is considered a severe central nervous system infectious disease with a high morbidity and mortality rate worldwide. The cerebrospinal fluid culture is the gold standard for the diagnosis of bacterial meningitis, but its detection rate is low due to antibiotic treatment Most patients had already received antibiotics before the lumbar puncture (van de Beek et al., 2016a; van de Beek et al., 2016b; McGill et al., 2016; Obaro, 2019). Viral encephalitis and meningitis are also common central nervous system infections; in particular, viral encephalitis can cause serious sequelae, leading to a heavy economic burden. The diagnosis of the viral infections of the central nervous system is often combined with routine cerebrospinal fluid examination, serology, and polymerase chain reaction (PCR) (McGill et al., 2017; Tyler, 2018; Venkatesan and Murphy, 2018). Microbial cultures require the use of different media and culture conditions, some of which are complex, time-consuming, and expensive. For example, *Bartonella*, which causes cat scratch disease in humans, grows slowly and takes 12–14 days in a culture (Lagier et al., 2015). PCR amplification techniques require specific primers that can lead to inaccurate results if the primers and the probe do not match the target nucleic acid sequence (Kuypers and Jerome, 2017).

Although one or several pathogenic bacteria can be detected by smear test, culturing, or PCR, there is still a lack of detection methods for rare pathogenic bacteria in clinical practice, so it is challenging to diagnose the etiology of CNS infections (Chen et al., 2021). The clinical diagnosis of mixed infections is very difficult. For example, mixed-strain infections in TB pose a huge challenge. The accurate diagnosis of a mixed infection plays a decisive role in clinical treatment, but traditional detection methods have certain limitations for the diagnosis of mixed infections (Tarashi et al., 2017). mNGS can detect all pathogenic microorganisms including bacteria, fungi, viruses, and parasites, especially for the cases that are most difficult to diagnose clinically, immunocompromised, and have mixed infections (Chiu and Miller, 2019). Traditional detection methods have very low diagnosis rates in patients with encephalitis. Epidemiological studies demonstrate that about 50% of the patients with encephalitis cannot obtain an etiological diagnosis. mNGS of nucleic acid extracted from cerebrospinal fluid or brain tissue plays a huge role in the diagnosis of central nervous system infections (Ramachandran and Wilson, 2020).

## Methods

### Study population

A retrospective cohort study was conducted on patients with central nervous system infections who were admitted to The First Affiliated Hospital of Zhengzhou University in Henan Province. The study period was between 1 January 2020 and 31 December 2020. We included patients who underwent mNGS examination at Henan Provincial Gene Hospital. The exclusion criteria included the following (van de Beek et al., 2016a): patients with incomplete clinical data (McGill et al., 2016), rejection of lumbar puncture or bloody cerebrospinal fluid (van de Beek et al., 2016b), and a lack of mNGS specimens.

The diagnosis of central nervous system infections includes etiological and clinical diagnoses. Patients who met the following 1–5 criteria underwent etiological diagnosis, and patients who met the following 1–4 criteria underwent clinical diagnosis : (1) Clinical manifestations:① changes in consciousness and mental state; ② symptoms of increased intracranial pressure; ③ meningeal irritation sign; ④ associated symptoms such as epilepsy and hyponatremia; and ⑤ symptoms of a systemic infection: symptoms and signs of the systemic inflammatory reaction such as the body temperature exceeding 38°C or falling below 36°C, leukocytosis, and rapid heart rate and breathing. (2) Clinical imaging: CT or MR may show diffuse cerebral edema, dural thickening and enhancement, or ventricular system dilatation; and enhanced imaging may show typical ring-enhanced space-occupying lesions and low-density purulent cavities. (3) Blood test: A routine complete blood count showed that white blood cells were higher than 10×10^9^/L and the proportion of neutrophils was more than 80%. (4) Lumbar puncture and general characteristics test of cerebrospinal fluid: ① lumbar puncture pressure was >200 mmH_2_O; ② cerebrospinal fluid turbidity showed yellow or typical purulence, if the formation of a package is clear and of transparent character; ③ the total white blood cell count in cerebrospinal fluid was >100–1,000×10^6^/L, and the multinucleated white blood cell count was >70%; ④ the cerebrospinal fluid glucose content decreased, with glucose <2.6 mmol/L, and the cerebrospinal fluid glucose/serum glucose ratio was <0.66; ⑤ the cerebrospinal fluid protein content was >0.45 g/L; ⑥ the elevation of CSF lactic acid had a certain reference value in the diagnosis of intracranial infections; and ⑦ molecular biological technology of cerebrospinal fluid: for patients with negative cerebrospinal fluid smear and culture and difficulties in diagnosis, PCR and other molecular biological technologies can be used to help identify the etiology. (5) The bacteriological examination of cerebrospinal fluid, surgical incision secretion, and surgical specimen was positive. Positive culture is the gold standard for diagnosis, but it is necessary to exclude specimen contamination, and negative cultures cannot be excluded.

A total of 94 patients with CNS infections were included in this study, of which 47 had intracranial infections, 22 had encephalitis, 17 had meningitis, 4 had meningoencephalitis, and 4 had brain abscesses. We also established the mNGS Professional Committee consisting of a panel of clinical experts who evaluated our cases and ensured the reliability of our data and the correctness of clinical diagnosis.

### Specimen collection

In clinical practice, patients with suspected CNS infections underwent lumbar puncture and imaging after obtaining informed consent. Cerebrospinal fluid samples were subjected to routine testing, including bacterial and fungal smears, acid-fast staining, bacterial and fungal cultures, and testing for a complete set of viruses. PCR was performed on cerebrospinal fluid samples from patients with suspected tuberculous encephalitis or meningitis. A patient with suspected cryptococcal meningitis was also tested for the cryptococcal antigen by staining with ink. mNGS of cerebrospinal fluid was detected in all patients.

In this study, we collected mNGS and microorganism cultivation information, as well as defined some time nodes. The following are some of the time nodes defined in the study: sample time, from the first day of hospitalization to the day of sample collection; turnaround time (TAT), from submission to mNGS or receiving microorganism culture report; and result time, from the first day of admission to receiving mNGS or a microbial culture report.

### Sample preparation, DNA extraction, library construction, and sequencing

At least 1 mL CSF samples were collected from patients according to the standard clinical procedure. DNA was extracted using the QIAamp^®^ UCP Pathogen Kit (Qiagen, Germany) according to the manufacturer’s recommendations. The extracted DNA samples were quantified using a Qubit fluorometer (Thermo Fisher Scientific, CA, United States).

DNA libraries were prepared using the TruePrep DNA Library Prep Kit V2 for Illumina^®^ (Vazyme, Nanjing, China) according to the manufacturer’s instructions in the manual. An Agilent 2100 Bioanalyzer (Agilent Technologies, Santa Clara, United States) was used for assessing library quality control. All libraries were pooled with other libraries using different index sequences and sequenced on an Illumina NextSeq 550Dx platform with a single-end 75-bp sequencing option. For each run, no template control (NTC) samples (nuclease-free H_2_O) were also pooled to monitor the reagent and laboratory background.

### Bioinformatic analyses

FASTQ format data were generated for each sample using bcl2fastq software (v2.20.0.422; parameters used: -barcode-mismatches 0 --minimum-trimmed-read-length 50). Adapting sequences and low-quality reads were removed using Cutadapt v2.10 (-q 25, 25 -m 50). The remaining high-quality reads were first depleted for human sequences by mapping to the human genome (hg38; https://hgdownload.soe.ucsc.edu/downloads.html#human) using bwa-mem2 v2.1 with default parameters; all unmapped reads were then aligned to the NCBI nt database (https://ftp.ncbi.nlm.nih.gov/genomes/) using BLAST+ v2.9.0 (-task megablast -num_alignments 10 -max_hsps 1 -evalue 1e-10). The alignments were required to be full-length with an identity of at least 95%. A customized Python script was used to identify species-specific alignments. Only the alignments that fulfilled the aforementioned criteria were used for further pathogen identification. The remaining microorganisms were defined as credible if the following criteria were met: (1) the microbe had at least three non-redundant mapped reads per 10 million raw sequence reads (except for the *Mycobacterium tuberculosis*); (2) for the *M. tuberculosis* complex (MTBC), due to the difficulty in detection, when at least one taxon-specific, high-quality aligned read was identified, the sample was reported as MTBC-positive; (3) the microbe was known to be potentially pathogenic in the given clinical context of each patient.

### Statistical analysis

SPSS 26.0 software was used for statistical analysis (IBM Corp, Armonk, NY, United States). Continuous variables with normal distributions were expressed as mean ± SD; continuous variables with non-normal distributions were presented as medians and interquartile ranges (IQRs). The Shapiro–Wilk test (*p* < 0.05) was used for continuous variables that were not normally distributed. The categorical variables were expressed as counts (no.) and percentages (%). The chi-squared test was used to compare the pathogen detection rate between mNGS and conventional methods. The sensitivity, specificity, positive predictive value, and negative predictive value were calculated using the culture method as the reference standard. *p* < 0.05 was considered statistically significant without multiple testing adjustment.

## Results

### Patients’ characteristics

The characteristics of all patients with central nervous system infections are summarized in Table 1. A total of 94 patients were enrolled in this retrospective study between 1 January 2020 and 3 December 2020. Of these, 60.6% (57/94) of the patients were male. The median age of the patients was 44 years (range 0.25–82). Among them, 31 patients were below 18 years and 21 patients were above 60 years. The details of the laboratory test data are given in Table 1, which include leukocytes, C-reactive proteins, procalcitonin, and cerebrospinal fluid. The hospital stay was less than 10 days for 13 people, 10–30 days for 44 people, and more than 30 days for 37 people, indicating that the hospital stay of patients with central nervous system infections was longer. At the time of discharge, 55 patients showed improvements, 20 patients were cured, five patients showed no improvement, two patients died, and 12 others. The median hospitalization cost was $20,918.9 (8472.6; 31721.6), with a high limit of $152,448.0 and a low limit of $1,323.

**TABLE 1:** Baseline characteristics of the participants.

Characteristic	Total (n = 94)
Age, median (IQR)	44 (12, 57)
Gender, no. (%)	
Male	57 (60.6)
Female	37 (39.4)
Laboratory test WBCs (10^9^/L), median (IQR)	10.9 (7.3, 15.3)
Procalcitonin (ng/mL), median (IQR)	0.16 (0.06, 0.58)
CRP (mg/L), median (IQR)	25.2 (3.3, 82.1)
CSF	
Protein (mg/L)	896.5 (408.5, 2662.8)
Chlorine (mmol/L)	121.0 (116.0, 128.3)
Glucose (mmol/L)	3.4 (2.7, 4.4)
CSF/blood glucose ratio	0.47 (0.23)
Nucleated cells (10^6^/L)	155.5 (30.25, 856.5)
Hospital days, no. (%)	
<10 days	13 (13.8)
10–30 days	44 (46.8)
>30 days	37 (39.4)
Outcome, no. (%) improved	56 (58.9)
Cured Unchanged Died Other hospitalization costs (dollar), median (IQR)	20 (21.1) 5 (5.3) 2 (2.1) 12 (12.6) 20918.9 (8472.6, 31721.6)

IQR, interquartile range; WBC, white blood cell; CRP, C-reactive protein; CSF, cerebrospinal fluid.

### Concordance between mNGS and conventional diagnostic testing

**FIGURE 1:**
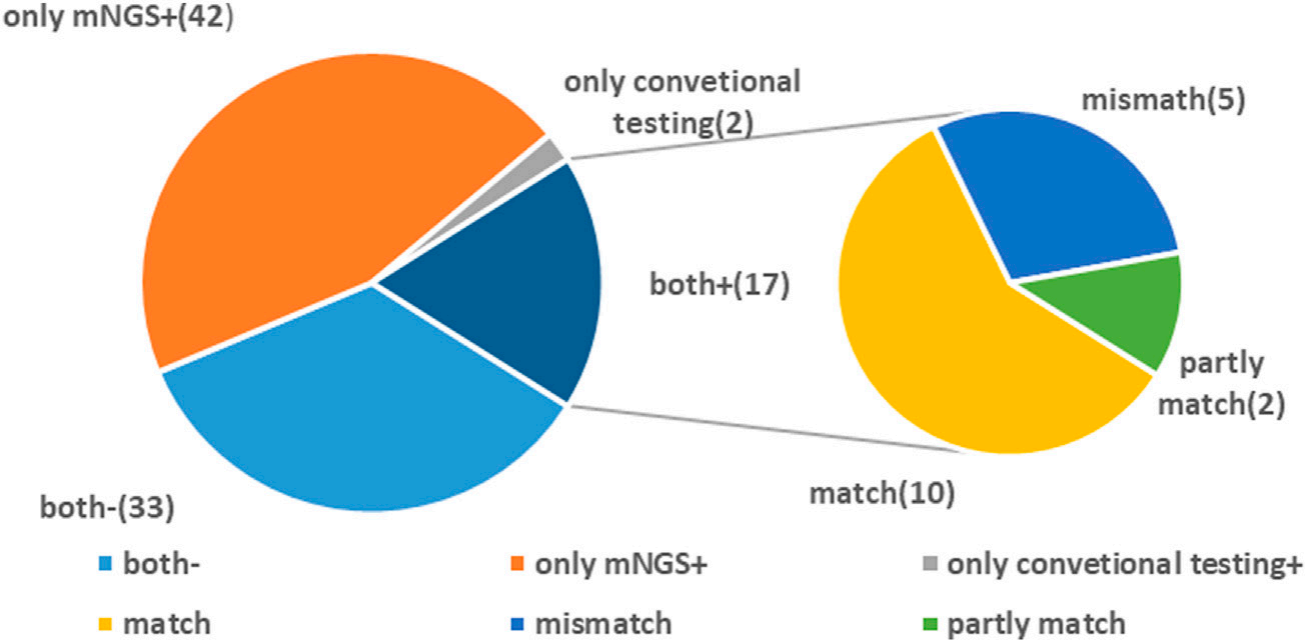
Concordance between metagenomic next-generation sequencing (mNGS) and conventional testing. Both +, both mNGS and traditional tests were positive; both −, both mNGS and traditional tests were negative; mNGS +, only the mNGS result was positive, while traditional test result was negative; only conventional testing +, only the traditional test result was positive, while mNGS was negative.

In this study, 17 cases were positive for mNGS and routine tests. The number of cases where pathogens were tested positive with mNGS alone was 42 while that with conventional methods alone was 2. Among the 17 double-positive cases, mNGS and conventional detection methods were completely consistent in 10 cases, partially consistent in two cases, and completely inconsistent in five cases. A total of 33 cases were negative for both routine testing and mNGS (Figure 1).

### Samples with positive CSF cultures

A total of 17 patients were found to have positive CSF cultures, among which one patient had fungal infection. Of these patients with positive CSF cultures, 13 patients developed results for resistant bacteria, including one fungal infection. Of the 17 positive CSF culture specimens, 10 cases were consistent with mNGS results. In 5 of the 17 patients with positive CSF cultures, the results were inconsistent with mNGS. Cerebrospinal fluid culture specimens of 14 cases showed *K. pneumoniae* (*Klebsiella pneumoniae*), while mNGS showed *Escherichia coli* (*E. coli*). In 17 cases, both mNGS and CSF cultures detected *K. pneumoniae*, but CSF also detected *Staphylococcus hominis* (*S. hominis*). In Case 20, CSF culture detected *Acinetobacter baumannii* (*A. baumannii*), while mNGS detected *K. pneumoniae*. In Case 36, CSF cultures detected *Providencia rettgeri* (*P. rettgeri*), while mNGS detected only human parvovirus B19. In Case 46, CSF cultures detected *Staphylococcus aureus* (*S. aureus*), while mNGS detected *Cryptococcus neoformans* (*C. neoformans*). Both CSF and mNGS detected *Stenotrophomonas maltophilia* (*S. maltophilia*) in Case 29, while mNGS also detected *Enterococcus faecium* (*E. faecium*) and the *Bacillus cereus* group. In addition to *K. pneumoniae* jointly detected by CSF cultures and mNGS in Case 34, mNGS also detected *Nocardia carnea* (*N. carnea*). In Case 68, *K. pneumoniae* was detected in CSF cultures but was negative in mNGS. In Case 73, the CSF culture detected *Moraxella osloensis* (*M. osloensis*), while mNGS was negative (Table 2).

**TABLE 2:** Samples with positive CSF cultures.

Patient ID	CSF culture result	mNGS result
C3	*A. baumannii*	*A. baumannii*
C6	*Staphylococcus*. *capitis*	*Staphylococcus*. *capitis*
C14	*K. pneumoniae*	*E. coli*
C17	*S. hominis* and *K. pneumoniae*	*K. pneumoniae*
C18	*K. pneumoniae*	*K. pneumoniae*
C20	*A. baumannii*	*K. pneumoniae*
C21	*A. baumannii*	*A. baumannii*
C29	*S. maltophilia*	*S. maltophilia* and *E. faecium*
C30	*K. pneumoniae*	*Bacillus cereus* group
C34	*K. pneumoniae*	*K. pneumoniae*
C35	*A. baumannii*	*K. pneumoniae* and *N. carnea*
C38	*P. rettgeri*	*A. baumannii*
C42	*S. aureus*	Human parvovirus B19
C45	*S. aureus*	*S. aureus*
C46	*C. neoformans*	*C. neoformans*
C68	*K. pneumoniae*	*C. neoformans*
C73	*M. osloensis*	Negative
		Negative

### mNGS positive results

**FIGURE 2:**
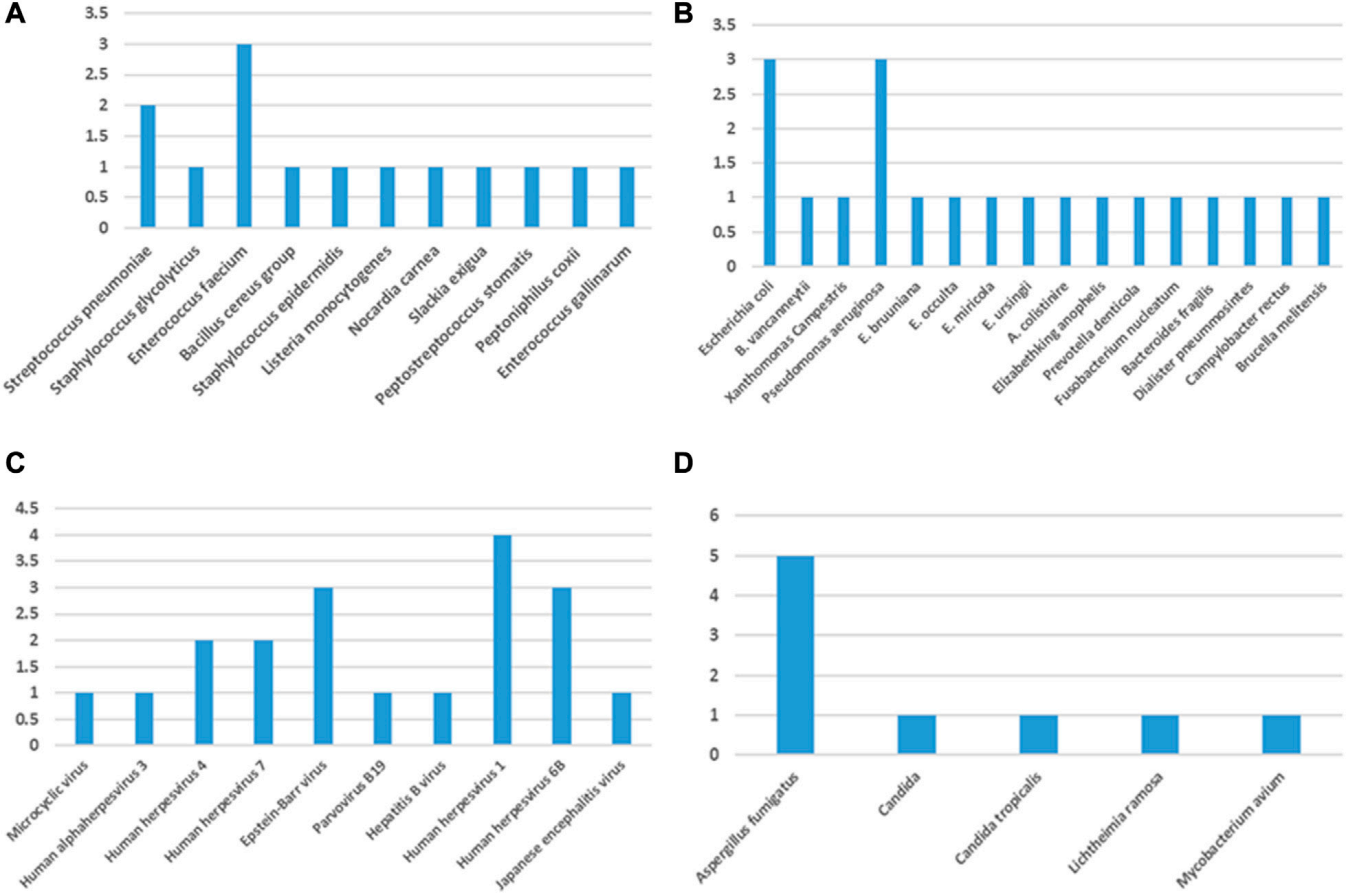
Species distribution of **(A)** Gram-positive bacteria, **(B)** Gram-negative bacteria, **(C)** viruses, and **(D)** fungi and other pathogens (*Mycoplasma*, *Chlamydia psittaci*, and *M. tuberculosis*) detected by mNGS.

**FIGURE 3:**
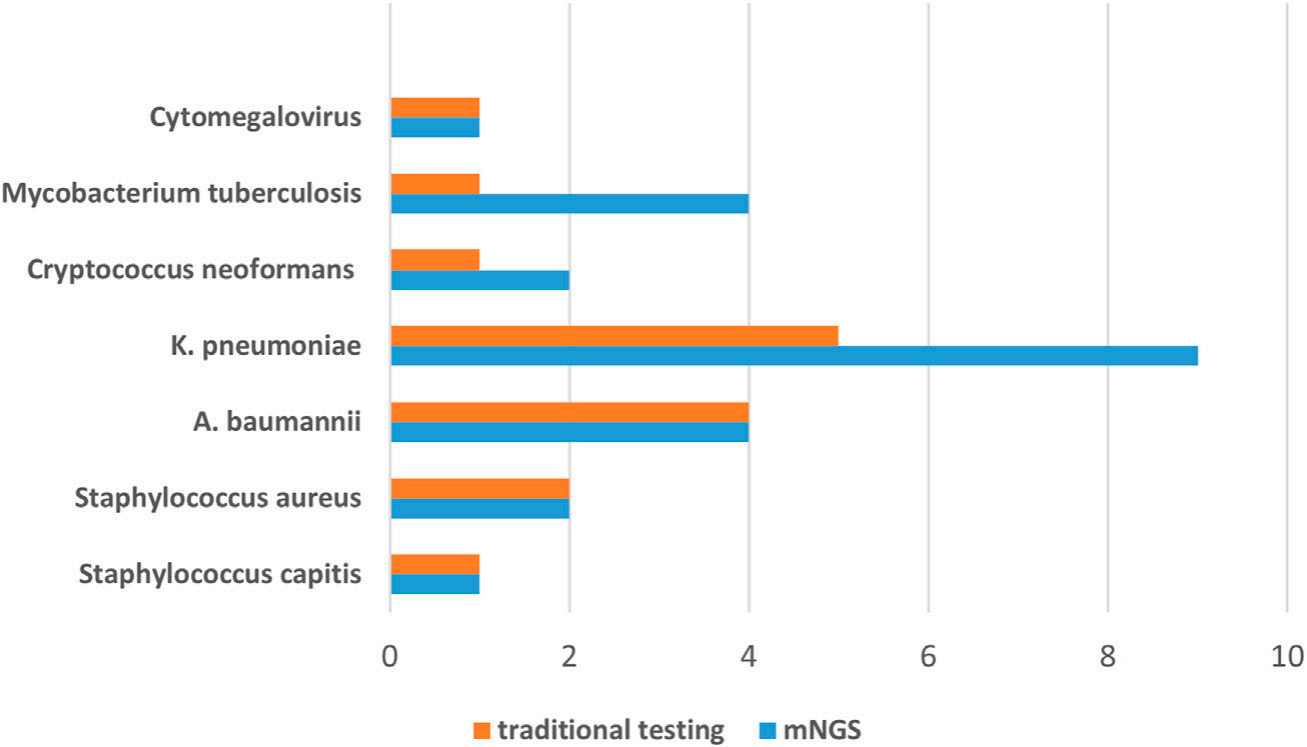
Pathogens detected by both conventional and mNGS methods.

In this retrospective study, 11 Gram-positive bacteria, 16 Gram-negative bacteria, 4 fungi, 10 viruses, and one special pathogen were detected by mNGS alone (Figure 2). The most detected Gram-positive bacteria was *E. faecium* (*E. faecium*; n = 3) and the most detected Gram-negative bacteria were *E. coli* (n = 3) and *Pseudomonas aeruginosa* (*P. aeruginosa*; n = 3). The most detected virus was human herpesvirus 1 (n = 4), and the most common type of fungus was *Aspergillus fumigatus* (*A. fumigatus*; n = 5). A total of seven pathogens, namely, *Staphylococcus capitis* (*S. capitis*), *Staphylococcus aureus*, *K. pneumoniae*, *A. baumannii*, *C. neoformans*, *M. tuberculosis*, and Cytomegalovirus (CMV), were detected by both conventional methods and mNGS (Figure 3). The most detected bacteria by mNGS are *K. pneumoniae* (n = 9), *A. baumannii* (n = 4), *P. aeruginosa* (n = 3), *E. faecalis* (n = 3), and *S. aureus* (n = 2). The most common fungi detected by mNGS were *Aspergillus fumigatus* (n = 5) and *Cryptococcus neoformans* (n = 2). The most common viruses detected by mNGS were the human herpesvirus (n = 11) and Epstein–Barr virus (EBV; n = 3). The most detected bacteria by traditional methods were *K. pneumoniae* (n = 5) and *A. baumannii* (n = 4), and the most common fungus was *C. neoformans* (n = 1). In the special pathogen detection, mNGS detected *M. tuberculosis* in four cases and *Mycobacterium avium* (*M. avium*) in one case, while conventional assays detected only *M. tuberculosis*. The results show that mNGS is superior to traditional methods in detecting both species and quantity of pathogens, especially fungi, mycobacteria, and viruses.

### Comparison of detection rates of mNGS and conventional tests

**FIGURE 4:**
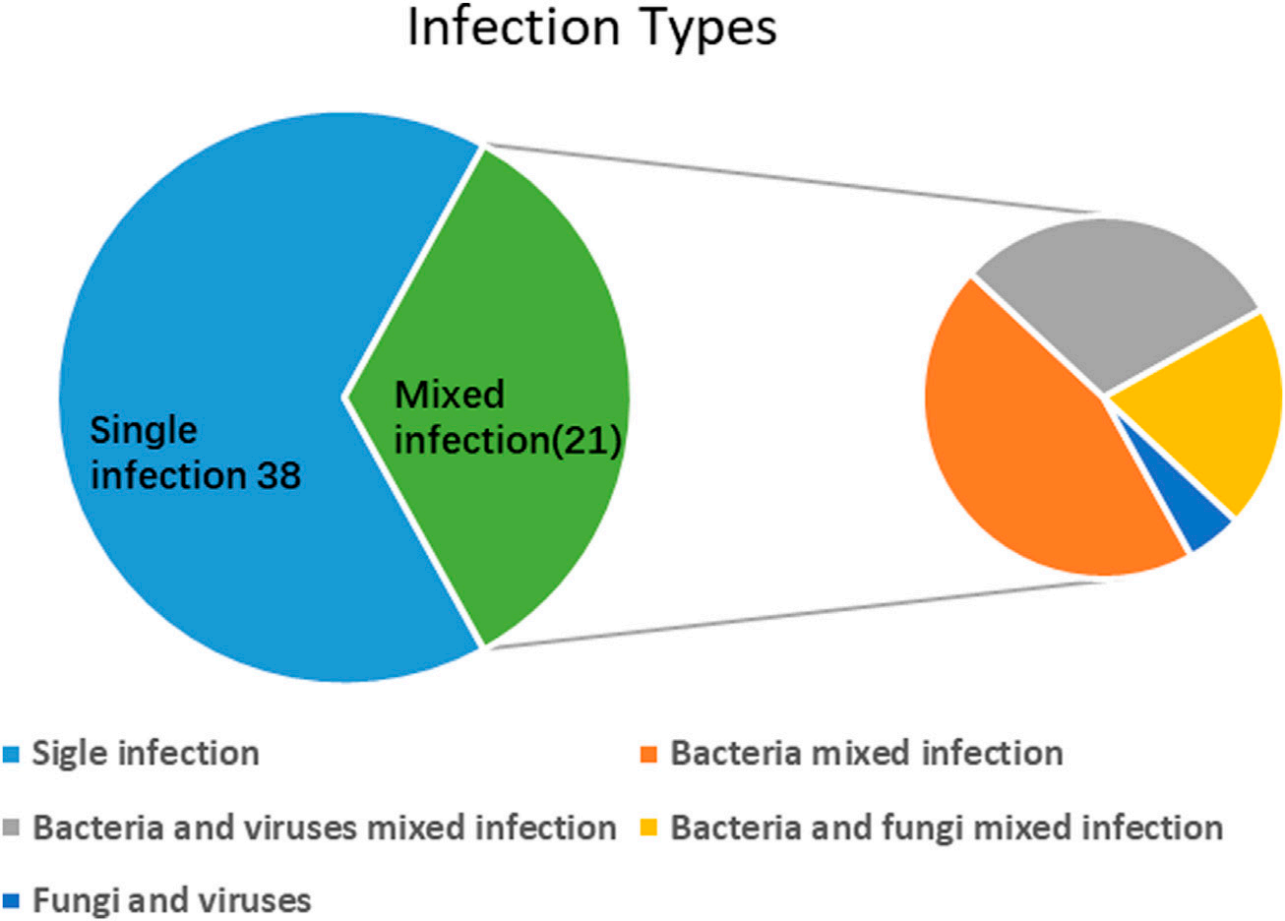
Mixed infections detected by mNGS and conventional tests.

The detection rate of mixed infections using mNGS alone was 19.1% (18/94). However, when combined with conventional tests, the detection rate increased to 22.3% (21/94). Only one case of mixed infection was detected by the culture. The most common type of mixed infection is bacterial mixed infection (n = 9), followed by bacterial and viral mixed infection (n = 7), bacterial and fungal mixed infection (n = 4), and fungal and viral infection (n = 1) (Figure 4).

A total of 28 (29.8%) cases with single-pathogen infections were detected by mNGS, including 17 bacterial, 12 viral, and 4 fungal infections, but only two cases (2%) with single-pathogen infections were detected by conventional testing. For single-pathogen infection, the detection rates of mNGS and conventional testing were statistically different (29.8% *vs* 2%; *p* < 0.05). In single-pathogen infection, eight cases were detected by mNGS and conventional assays. In this study, we define mixed infections as one patient infected with more than one pathogenic microbe (including bacteria, fungi, and viruses). In this study, mNGS was superior to traditional detection methods in detecting both single and mixed infections.

### Diagnostic performance of mNGS

**FIGURE 5:**
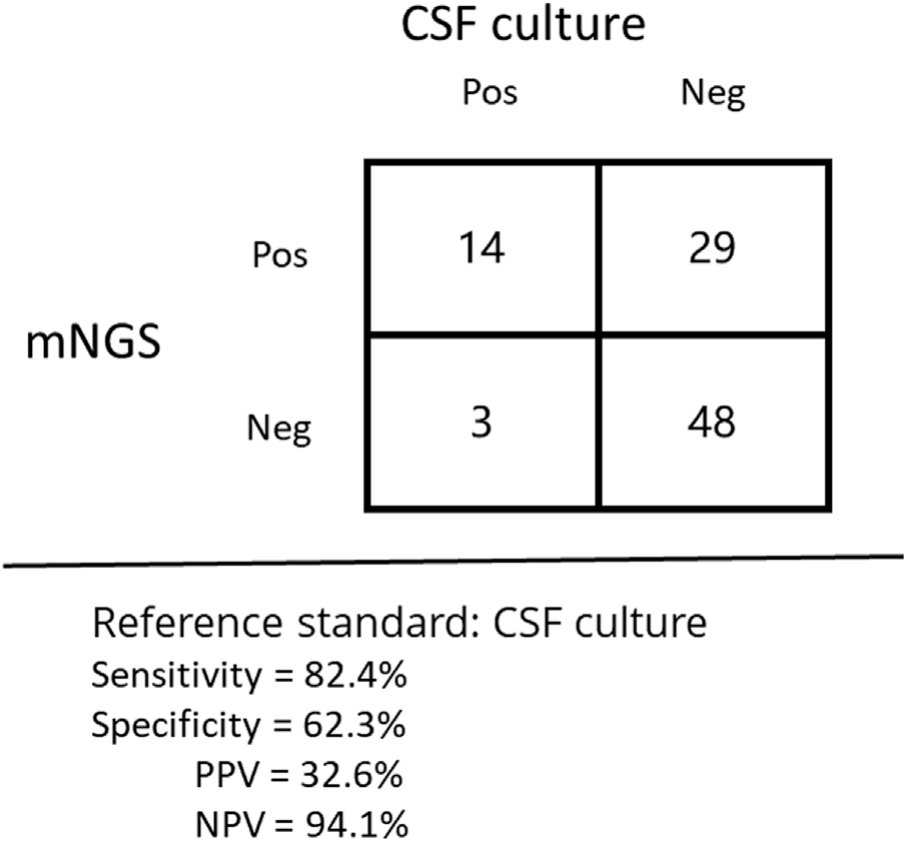
Performance of different methods for the diagnosis of tuberculous meningitis.

Using the culture method as the reference standard, the sensitivity and specificity of mNGS in the diagnosis of CNS infections were 82.4% (14/17) and 62.3% (48/77), and the positive predictive value and negative predictive value were 32.6% and 94.1% (without virus, Figure 5), respectively. In 94 samples, the positive rate of pathogens detected by the mNGS method was 60.6% (57/94), while the positive rate of pathogens detected by the traditional method was 21.3% (19/94), indicating a statistically significant difference between the two methods (*p* < 0.01). In general, mNGS is superior to traditional methods in the diagnosis of CNS infections.

### The effect of positive mNGS on clinical therapeutic effects

We analyzed the impact of positive mNGS reports on clinical treatment. The influence on clinical treatment can be divided into five categories: (1) providing etiological evidence for unidentified infections, mNGS plays an active role in guiding clinical treatment; (2) consistent with the clinician’s judgment on infection (the clinician had made an empirical diagnosis in combination with culture or other tests before the mNGS results were available), mNGS played a role in validation; (3) after mNGS results were informed, the family members refused treatment or transferred to another hospital, which hinders further treatment; (4) the mNGS results were not consistent with clinical symptoms, and the possible causes were considered contamination; and (5) when the patient was discharged, it was not possible to determine whether the mNGS results were pathogenic bacteria. mNGS provided diagnosis for eight cases of infection without clear etiology, of which six cases were due to the cerebrospinal fluid culture results rather than mNGS results, but the culture results of these six cases were consistent with mNGS. Consistent with the clinician’s judgment on infection, mNGS played a role in the verification of 20 cases. After the mNGS results were informed, 25 patients could not receive further treatment because their family members refused treatment or transferred to another hospital. The mNGS results were not consistent with clinical symptoms in two cases, which might be considered contamination. At discharge, it was not possible to determine whether the mNGS results were pathogenic in one patient (Table 3).

**TABLE 3:** Effect of positive mNGS on clinical therapeutic effect.

Type no. (%)	Total (n = 57)
Positive guidance	8 (14)
Verified effect	20 (35.1)
Not used	25 (43.9)
Possible contamination	3 (5.3)
Indeterminate	1 (1.8)

It is worth mentioning that there are several special cases. In one patient, the mNGS results of CSF samples were consistent with the results of blood culture, both of which were *E. coli*, but the CSF culture showed *K. pneumoniae*, and the clinician believed that the CSF culture was likely to be contaminated. In one patient, the CSF culture was positive for *A. baumannii*, the mNGS results were positive for *K. pneumoniae*, and the blood culture was positive for *Burkholderia cepacia*, but the clinician treated the patient according to the blood culture results. *Brucella melinii* was detected by mNGS, and the patient also had tuberculous meningitis. Both diseases were treated clinically. When the patient was discharged from the hospital, it was still uncertain whether she had been infected with *Brucella melinii*.

## Discussion

In this study, we enrolled 94 patients with central nervous system infections, and CSF was sequenced by mNGS. We compared the results of mNGS and traditional testing methods and analyzed the diagnostic performance of mNGS and the role of positive mNGS results in guiding clinical treatment. Consistent with previous studies, mNGS is superior to conventional methods in diagnosing mixed infections and infections with specific pathogens. Furthermore, mNGS developed a faster turnaround time (TAT) than conventional methods (Xing et al., 2022). Traditional testing methods mainly rely on pathogen-specific testing, but mNGS can overcome this drawback by analyzing all nucleic acids in the sample. The detection rate of mNGS in our study was 60.8%, which was basically consistent with 29%–60% reported in the literature (Wilson et al., 2019). The combination of two detection methods shows that mNGS not only has advantages in diagnosing CNS infections but also shows a higher diagnosis rate when combined with traditional detection methods. Particularly, during the diagnosis of rare pathogens of central nervous system infections, it provides a new perspective (Zhan et al., 2021; Zhou et al., 2021).

Culture is generally considered the gold standard for bacterial pathogens but stringent culture conditions and prior antibiotic treatment have hampered its effectiveness. In this study, the CSF culture detection rate was only 18%, much lower than the 50% detected by mNGS for bacteria. The low culture rate of CSF is most likely due to the use of antibiotics prior to the culture. Traditional bacterial cultures lasted around 3–7 days, while fungi and tuberculosis grew longer resulting in a delay in establishing the diagnosis. The average TAT of mNGS in this trial was 1.4 days. *S. pneumoniae* and *Neisseria meningitidis* are the most common pathogens causing bacterial meningitis, which have caused a serious impact on public health security worldwide (Koelman et al., 2019). However, in our study, *K. pneumoniae* and *A. baumannii* were the main pathogens. This may be due to the differences in the types of pathogens in different areas. With the emergence of carbapenem-resistant strains, the treatment of bacterial meningitis caused by *K. pneumoniae* and *A. baumannii* poses challenges, with high mortality and severe neurological sequelae. It has been reported that *Listeria monocytogenes* has become one of the common pathogens causing bacterial meningitis, but routine detection methods are not sensitive. For *Listeria* infection, the diagnosis remains difficult (Zhang et al., 2021). Notably, both *P. rettgeri* and *M. osloensis*, which were cultured by traditional methods, were not detected in mNGS. *P. rettgeri* is an opportunistic pathogen that causes fewer reported infections, but the bacterium is naturally resistant to many antibiotics (Mc Gann et al., 2021).

Viruses are also a common cause of CNS infections, the most common viral infection being the human herpes simplex virus. Viral encephalitis or meningitis can cause severe mortality and disability (Lee et al., 2021). Polymerase chain reaction (PCR) is commonly used to detect viruses, but it requires specific primers. Therefore, this requires clinicians to make a judgment on the kind of virus that the patient is infected with, and mNGS can overcome this disadvantage. In this study, in addition to common herpesviruses, Cytomegalovirus, and Epstein–Barr virus, Japanese encephalitis virus (JEV) was also detected by mNGS. Out of the two confirmed cases of JEV, mNGS detected JEV in only one case and *M. avium* in the other. Since JEV is an RNA virus that requires reverse transcription before sequencing, it may reduce the number of DNA fragments, resulting in a lower detection rate. JEV can cause severe damage to the central nervous system, and we recommend a mNGS test for JEV with both serotype and PCR-negative (Howard-Jones et al., 2022).

In this study, the number of cases of tuberculous and fungal encephalitis or meningitis was small, but the detection rate of mNGS was still higher than that of conventional diagnostic methods. Of all TB infection types, tuberculous meningitis is the most lethal form of TB, with a mortality rate of up to 50%. The diagnosis of tuberculous meningitis is difficult because *M. tuberculosis* is difficult to detect in cerebrospinal fluid, and delayed diagnosis and drug resistance are two major problems in tuberculous meningitis (Soria et al., 2021). The diagnostic rate of tuberculous meningitis in this study was 62.5% (5/8), which was consistent with the literature reports. NGS technology can rapidly detect MTB in cerebrospinal fluid and detect drug resistance genes. NGS can be recommended as a first-line diagnosis of tuberculous meningitis (Lin et al., 2021). Fungal infections of the central nervous system have a high mortality rate, especially in immunocompromised individuals (Sharma et al., 2022). In our study, there were five cases of *A. fumigatus*, two cases of *C. neoformans*, one case of *P. racemosus*, one case of *Candida*, and one case of *C. tropicalis*. Among the two cases of C*. neoformans*, one case was diagnosed as a mixed infection of *Cryptococcus neoformans* and *Staphylococcus aureus* by mNGS combined with CSF culture. The fungal cell wall is thick and easy to break, and DNA fragments are difficult to obtain, which may affect the detection rate of fungi. When CNS infections are more complex, we recommend a combination of mNGS and traditional testing methods.

Central nervous system infections impose a heavy financial and mental burden on patients due to the risk of severe neurological sequelae and have a high mortality rate (Acuña et al., 2022; Chala et al., 2022). In the study, the median cost of hospitalization for patients with CNS infections was $20,918.99 (8472.6; 31721.6), and the maximum cost of hospitalization was $151,792.8. At the same time, we found that 26.6% (25/94) of the patients gave up treatment because the disease was too severe even if the infectious pathogen was detected. Therefore, it is reasonable to recommend mNGS as one of the auxiliary tests for early diagnosis. Early diagnosis and early treatment can reduce the burden and bring hope to patients.

### Limitations

Our study also has some limitations. This study is a retrospective study, and therefore, the data collection is all retrospective. We need rigorous large-scale prospective studies to verify the experimental results. Moreover, the nucleic acid extraction efficiency of mNGS is very important for mNGS results, and in future studies, the extraction efficiency of various kits will be compared to determine the highest diagnostic value of mNGS.

## Conclusion

In conclusion, mNGS plays a vital role in the diagnosis of central nervous system infections, and we hope that the diagnostics of this new technology can guide clinical treatments to reduce mortality and disability rates in related patients.

## Data Availability

The datasets presented in this study can be found in online repositories. The names of the repository/repositories and accession number(s) can be found in the article/Supplementary Material.
